# Elevated Soluble Suppressor of Tumorigenicity 2 Predict Hospital Admissions Due to Major Adverse Cardiovascular Events (MACE)

**DOI:** 10.3390/jcm12082790

**Published:** 2023-04-09

**Authors:** Dongqing Chen, Rossana Untaru, Glykeria Stavropoulou, Bahador Assadi-Khansari, Conagh Kelly, Amanda J. Croft, Stuart Sugito, Nicholas J. Collins, Aaron L. Sverdlov, Doan T. M. Ngo

**Affiliations:** 1 School of Biomedical Sciences and Pharmacy, The University of Newcastle, Callaghan, NSW 2308, Australia; 2 Hunter Medical Research Institute, New Lambton Heights, NSW 2305, Australia; 3 School of Medicine and Public Health, The University of Newcastle, Callaghan, NSW 2308, Australia; 4 Cardiovascular Department, John Hunter Hospital, Hunter New England Local Health District, Newcastle, NSW 2305, Australia

**Keywords:** biomarker, sST2, MACE, cardiovascular, outcomes

## Abstract

The role of soluble suppression of tumorigenicity (sST2) as a biomarker in predicting clinical outcomes in patients with cardiovascular diseases (CVD) has not been fully elucidated. In this study, we sought to determine the relationship between sST2 levels and any unplanned hospital readmissions due to a major adverse cardiovascular event (MACE) within 1 year of first admission. Patients (n = 250) admitted to the cardiology unit at John Hunter Hospital were recruited. Occurrences of MACE, defined as the composite of total death, myocardial infarction (MI), stroke, readmissions for heart failure (HF), or coronary revascularization, were recorded after 30, 90, 180, and 365 days of first admission. On univariate analysis, patients with atrial fibrillation (AF) and HF had significantly higher sST2 levels vs. those who did not. Increasing levels of sST2 by quartiles were significantly associated with AF, HF, older age, low hemoglobin, low eGFR, and high CRP levels. On multivariate analysis: high sST2 levels and diabetes remained as risk predictors of any MACE occurrence; an sST2 level in the highest quartile (Q4: >28.4 ng/mL) was independently associated with older age, use of beta-blockers, and number of MACE events within a 1 year period. In this patient cohort, elevated sST2 levels are associated with unplanned hospital admission due to MACE within 1 year, independent of the nature of the index cardiovascular admission.

## 1. Introduction

Cardiovascular disease (CVD) remains the leading cause of death and disability in the world [1]. Chronic CVD is characterized by frequent exacerbations requiring readmissions, which in themselves are a marker of poor outcome [2], and additionally contribute to the majority of healthcare costs related to CVD [3]. Thus, the major adverse cardiovascular event (MACE) rate is commonly used in clinical trials as a main composite outcome measure of an intervention strategy. Ability to predict MACE, with a view to being able to intervene and treat, can form a foundation in our efforts to reduce poor CVD outcomes. In this space, biomarkers are an attractive and emerging tool to enhance the ability of the clinician to identify the patients at highest risk and focus management to prevent the occurrence of MACE. While there are currently multiple biomarkers and CV risk factors, such as cholesterol levels, troponin, and brain-natriuretic peptides (BNP), their predictive values for MACE in the era of personalized medicine are of only limited usefulness [4], and many patients with CVD have no conventional risk factors for the prediction of MACE [5]. There is a clear unmet clinical need to identify biomarkers that specifically underpin the key cardiovascular pathological processes to allow better predictive ability for MACE.

Suppressor of Tumorigenicity 2 (ST2) is a member of the interleukin-1 (IL-1) receptor family that functions as a critical effector molecule of T helper type 2 cell (Th2) responses and has 2 subtypes: soluble ST2 (sST2) and transmembrane tST2. ST2 and its ligand interleukin-33 (IL-33) are expressed on cardiac myocytes, fibroblasts, mast cells, eosinophils, and Th2 lymphocytes, and can be induced in macrophages [6]. ST2 has been shown to mediate cardiomyocyte-specific effects and is associated with activation of inflammation in the pathophysiology of cardiac damage. Myocardial stress and injury results in the upregulation of ST2 levels [7]. Inhibition of the interaction between IL-33 and tST2 leads to a reduction of myocardial fibrosis, cardiomyocyte hypertrophy, and apoptosis [8,9], while sST2 removes IL-33 from the circulation, promoting cardiac hypertrophy, fibrosis, and ventricular dysfunction [8]. Additionally, sST2 has been shown to be predictive of both the diagnosis of acute decompensated heart failure (ADHF) and of ADHF mortality; however, the predictive value of sST2 in other CVD states is less well studied, and its ability to predict MACE has not has not been evaluated [10].

Unplanned hospital admissions due to a major adverse cardiovascular event (MACE) are associated with significant financial impact on the health system, and are generally associated with poorer outcomes for the patient [2]. In this study, we evaluated the relationship between sST2 levels and MACE in patients admitted to a tertiary hospital cardiology unit. We hypothesized that patients with high sST2 levels are more likely to be at risk of a hospital admission due to MACE over 1-year period.

## 2. Materials and Methods

### 2.1. Study Cohort and Design

The Hunter New England region of New South Wales, Australia, covers an area of over 130,000 km^2^, and has a population of approximately 960,000, of whom approximately 45% live in metropolitan areas and 55% in regional or rural settings. John Hunter Hospital is the only major tertiary metropolitan teaching hospital in the district.

In this prospective longitudinal cohort study (n = 250), consecutive patients who presented to the cardiology unit at the John Hunter Hospital between December 2018 and February 2019 were recruited. Inclusion criteria were age ≥ 18, confirmed cardiovascular diagnosis requiring admission (as deemed by the treating team), and the ability to provide written informed consent. Exclusion criteria were the absence of a confirmed cardiovascular diagnosis and the inability to give written informed consent.

At recruitment, patients’ demographics, past medical history, and baseline pharmacological treatments were obtained from patients and their medical records. All patients had a baseline evaluation which included fasting blood samples for routine biochemistry, hemoglobin, lipid profile, plasma glucose, and soluble ST2 levels (measured by specific enzyme-linked immunosorbent assays (ELISA) kits (R&D systems, Minneapolis, MN, USA)).

Major adverse cardiac events (MACE) were defined as the composite of total death; myocardial infarction (MI), stroke, heart failure readmission; readmission requiring coronary revascularization, (including percutaneous coronary intervention and/or coronary artery bypass grafting) [11]. Data regarding each patient’s MACE were prospectively collected from the medical records at 30, 90, 180 days, and 1-year post admission.

Outcome data and comorbidities were further verified via the Cardiac and Stroke Outcomes Unit database, which has previously been described [12], and included hospital readmissions, emergency department presentation, in-patient death, and all-cause mortality, obtained from the state Births Deaths and Marriage Register. This database records all admissions to all public hospitals within the health district based on ICD (International Statistical Classification of the Diseases and Related Health Problems) codes [12]. Comorbidities were verified from any ICD-10 code recorded during the hospital admission when the patients were recruited to the study. Respective ICD-10 codes, as the reason for admission/presentation, for the above events described above were used to determine MACE (I21.xx–I24.xx; I50.x; I60–I69) for all subsequent hospital presentations. In-patient death and all-cause mortality were obtained from the state Births Deaths and Marriage Register as part of MACE.

All subjects gave their informed consent for inclusion before they participated in the study. The study was approved by the Human Research Ethics Committee of Hunter New England Local Health District (reference number: 2018/ETH00125).

### 2.2. Soluble ST2 Assay

Blood samples were obtained from all study participants using EDTA-containing tubes. Plasma samples were centrifuged immediately and stored at −80 °C until assayed. Plasma concentrations of sST2 levels were analyzed using the R&D Quantikine (R&D systems, Minneapolis, MN, USA) sandwich enzyme-linked immunosorbent assay (ELISA) technique. Assay calibration was performed according to the manufacturer’s recommendations, with values normalized to a standard curve.

### 2.3. Statistical Analysis

Baseline patient clinical characteristics were compared using independent sample t-test for parametric, and the Mann–Whitney U test for non-parametric data. Chi-square tests were performed for categorical variables. Soluble ST2 concentrations in patients with versus without hospital admissions due to MACE within 1 year were compared using the Mann–Whitney U test. All non-parametric continuous variables were log-transformed prior to linear regression analyses. All of the patient’s comorbidities and other risk factors were compared by sST2 quartiles by using the Kruskal–Wallis H test or Chi-square test, as appropriate. Stepwise backward logistic regression multivariable analyses were performed to establish predictors of (i) MACE events; and (ii) highest ST2 levels (by quartile)—both adjusted for age, gender, BMI, diabetes, hypertension, history of ischemic heart disease, heart failure, diabetes, atrial fibrillation, and use of medications including statins, antiplatelets, beta-blockers, ACE inhibitors, angiotensin receptor blockers (ARBs), and angiotensin receptor-neprilysin inhibitors (ARNI). The models were also re-run in a forward stepwise approach, limiting the number of variables at each step to ≤5 (starting with the most significant on univariate analyses). Kaplan–Meier analysis was used to examine time to MACE stratified by the sST2 level quartiles and log-rank (Mantel–Cox) test was used to determine significant difference between survival curves. All analyses were performed using IBM SPSS statistics software, version 26 (IBM, Armonk, NY, USA), and *p* < 0.05 was considered statistically significant.

## 3. Results

### 3.1. Patient Characteristics

Demographics and baseline characteristics of patients are presented in Table 1. The main reasons for admission were coronary artery disease, including ST-elevation myocardial infarction (STEMI; n = 45 (18%)), non-ST-elevation myocardial infarction (NSTEMI; n = 61 (24.4%)), percutaneous coronary procedures/acute coronary syndrome (n = 64 (26.6%)) heart failure (n = 43 (17.2%)), and atrial fibrillation (n = 7 (2.8%)). These are representative of patients with high-risk cardiovascular events presenting to cardiology unit. Approximately 90% of patients in this cohort had history of ischemic heart disease with multiple concomitant co-morbidities, as reflected in baseline medical therapy. The number and percentage of MACE for each of the comorbidity types is presented in Supplementary Table S1. Only history of diabetes was significantly associated with subsequent MACE (*p* = 0.001). Out of a total of 250 patients, 125 (50%) patients developed at least one MACE, and 58 (23%) patients developed multiple MACE within 1 year of follow up.

### 3.2. Univariate Analyses

On univariate analyses, male patients, as well as those with a history of HF and AF, had significantly higher sST2 levels vs. females and patients without history of HF and AF, respectively (Table 2). Comparisons of the sST2 levels in patients with or without comorbidities are shown in boxplot graphs (Supplementary Figure S1). Furthermore, increasing age and CRP levels were associated with elevated sST2 levels (Table 3). Patients who had at least one MACE were older compared to those without MACE during follow up (Table 4).

At enrolment all patients were stratified by serum sST2 level quartiles (Q1: <11.1 ng/mL; Q2: 11.1–16.8 ng/mL; Q3: 16.8–28.4 ng/mL; Q4: >28.4 ng/mL): higher sST2 level quartiles were significantly associated with AF, HF, older age, lower hemaglobin (Hb), low eGFR, and high CRP levels (Table 5).

**Figure 1:**
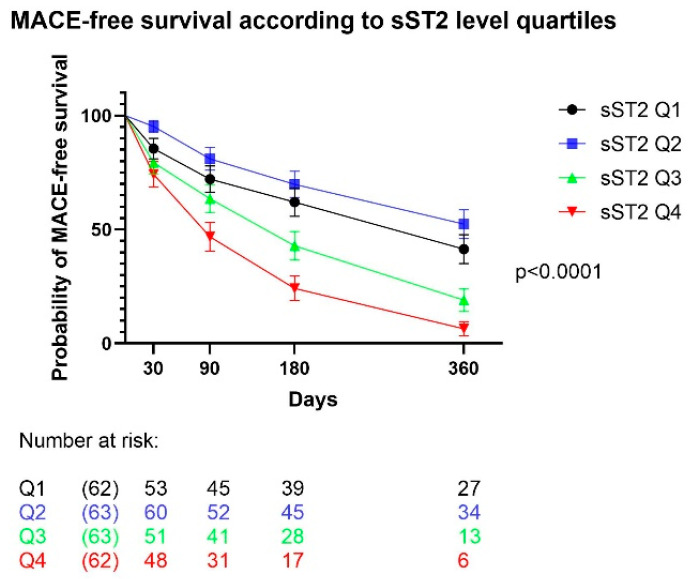
MACE-free survival according to sST2 level quartiles.

Kaplan–Meier analysis was performed to determine MACE-free survival according to sST2 level quartiles (Figure 1). The MACE-free survival was significantly worse in patients with sST2 levels in 2 highest quartiles (Q3 & Q4; *p* < 0.0001).

### 3.3. Multivariable Analyses

We performed backward logistic regression analyses using two models:

Model 1: predictors for any unplanned MACE admissions adjusted for age, gender, BMI, hypertension, dyslipidemia, history of ischemic heart disease, heart failure, diabetes mellitus (DM), atrial fibrillation, and use of medications including statins, antiplatelets, beta-blockers, ACE inhibitors, ARBs, and ARNI. High levels of sST2 (β = 1.02, *p* = 0.042, CI = (1.0–1.03)) and diabetes (β = 2.88, *p* = 0.001, CI = (1.6–5.2)) remained an independent risk for predictors of MACE occurrence (Table 6).

Model 2: predictors of highest sST2 quartile adjusted for number of MACE, as well as age, gender, BMI, DM, hypertension, dyslipidemia, history of ischemic heart disease, heart failure, atrial fibrillation, and use of medications including statins, antiplatelets, beta-blockers, ACE inhibitors, ARBs and ARNI. The highest sST2 quartile was independently associated with age (β = 1.03, *p* = 0.022, CI = (1.0–1.1)); use of beta-blockers (β = 2.27, *p* = 0.033, CI = (1.1–4.8)); and number of MACE (β = 1.35, *p* = 0.007, CI = (1.1–1.7)) (Table 7).

Re-running models 1 and 2 in a forward stepwise approach (i.e., adding the variables in one at a time starting with the most significant and limiting to ≤5 variables at each step), did not significantly alter the output of the model.

## 4. Discussion

In our study, for patients with established CVD who were admitted to a cardiology unit, a high level of sST2 was found to be predictive of hospital readmission due to MACE within 1 year. These findings extend the clinical usefulness of sST2 beyond just heart failure or CAD to the potential for predicting hospital readmissions for patients with a wide range of cardiovascular disease states.

ST2 is markedly upregulated upon the stimulation of cardiomyocytes with mechanical strain [7]. It was identified that ST2 exerts actions on the cardiovascular systems by interacting with its functional ligand IL-33 [8,13]. Upon tissue damage, IL-33 is expressed by stromal cells and signals to local immune cells [14]. Generally, the induction of inflammation leads to the activation of the IL-33/ST2 axis, resulting in a positive feedback loop that increases the production of proinflammatory cytokines/chemokines [15]. However, in the context of cardiovascular pathophysiology, the intact IL-33/ST2 pathway is cardioprotective: treatment with exogenous IL-33 reduces hypertrophy, while the genetic deletion of the ST2 membrane receptor abolishes the beneficial anti-fibrotic and anti-hypertrophic effect of IL-33 [13]. Soluble ST2 (sST2), a soluble secreted truncated form of ST2L, acts as a decoy receptor, binding and inhibiting IL-33 [8]. Therefore, elevated levels of sST2 in circulation attenuate the systemic effects of IL-33. In an animal study, ApoE (-/-) mice treated with soluble sST2 developed significantly larger atherosclerotic plaques in the aortic sinus compared to control mice [16]. Additionally, sST2 was found specifically expressed in arterial endothelial cells, and plays a role in the progression of atherosclerosis [17]. These results suggested potential underlying mechanism for sST2 serves as a marker of plaque burden, and to predict future cardiovascular events.

The predictive value of sST2 has been shown in previous studies in patients with STEMI [18,19,20], NSTEMI [18,20,21], and HF [22,23,24,25]. In the TIMI-ENTIRE trial, elevated sST2 levels in 810 patients with STEMI were correlated with mortality and onset or worsening of heart failure within 30 days post STEMI [19]. The CLARITY-TIMI 28 trial [26] built upon these results, finding that elevated sST2 levels at the time of presentation independently predicted major cardiovascular events and cerebrovascular events within 1 year of PCI. A prognostic value of sST2 has also been evaluated for longer-term follow-up. Elevated sST2 levels were recently found to be associated with markedly increased risks of MACE in patients with a stable CAD who were followed up for over 2 years [27]. Elevated sST2 concentrations alone or in combination with other biomarkers, such as cardiac troponin and natriuretic peptides, have also been shown to be predictive of new-onset HF, all-cause mortality, and/or HF hospitalizations [25].

Our study further investigated the prognostic value of sST2 in predicting future hospital admission due to MACE within 1 year of follow up in a cohort of patients admitted to hospital with various forms of CVD, including acute coronary syndromes, heart failure, and atrial fibrillation in the presence of other multiple cardiovascular comorbidities and risk factors. Our multivariable findings confirm that high levels of sST2 are associated with increasing incidence of MACE in a more heterogenous cohort of CVD patients. Conversely, 1 year hospital readmissions due to MACE were independently associated with higher sST2 levels. In fact, sST2 levels and DM were the only two significant predictors of 1-year MACE in this patient cohort. This suggests that sST2 levels are likely to represent a “unifying” risk stratification marker in a heterogenous cohort of patients with established CVD or a high CV risk factor burden.

Diabetes has been extensively shown to be a major cardiovascular risk factor and can directly lead to adverse cardiac outcomes through a wide variety of mechanisms, including increased inflammatory activation, oxidative stress, abnormal myocardial substrate metabolism, enhanced myocardial fibrosis and hypertrophy, and the generation of toxic advanced glycation end products [28]. The landmark randomized, long-term follow up Framingham Heart studies found that patients with diabetes had a two times higher risk of developing MACE than those without diabetes [29]. The ADVANCE study [30] and the EMPA-REG OUTCOME trial [31] showed that type 2 diabetic patients receiving glucose-lowering agents reduced their risk of MACE by 10–14%. Importantly, more of the recent studies demonstrated the prognostic value of sST2 in patients with diabetes: sST2, Hs-cTnI, and NT-proBNP are independently associated with mortality and onset of MACE in patients with diabetes; specifically, higher levels of sST2 led to an almost three-fold increase in the risk of death in a 15 year follow-up [32]. Additionally, sST2 remained a predictor of MACEs and mortality in patients with coronary artery disease, both with and without diabetes [33], as well as in patients with acute coronary syndrome and diabetes [34]. Consistent with the extensive prior literature, we found that, in our patient cohort with existing CVDs, both sST2 levels and diabetes mellitus were strong predictors of future MACE. In addition, high levels of ST2 did not associate with diabetes, which indicates that sST2 could be a good prognostic biomarker for MACE in patients with CVDs, regardless of diabetic comorbidity. 

Some of the study limitations include the lack of serial measurements of sST2 to predict patient outcomes. Sustained elevated levels of biomarkers, such as galectin-3 levels, have been shown to be an independent predictor of all-cause mortality or readmission for HF during 1-year follow-up [35]. It is possible that the combination of sST2 with other cardiac biomarkers, such as hs-Troponin and/or NT-proBNP, could be more sensitive and specific for predicting the risks of hospitalisation; however, we did not have NT-proBNP or hs-Troponin levels for all the patients, as the measurements of NT-proBNP or hs-troponin were not clinically indicated for all patients. Similarly, as only 28% of patients had heart failure and 27% had diabetes, neither iron studies nor HbA1c levels are available for a large proportion of this cohort. Additionally, while echocardiography is recommended for most of these patients, consistent with previous real-world data, inpatient echocardiography is severely underutilized [36]. Our study, while prospective, was an observational real-world study, and we did not mandate any investigation or management changes beyond what the treating clinicians chose to implement or perform. As a result, only 55% of patients in our study had an echocardiogram, which does not permit the use of echocardiographic data in analyses, as it is likely to introduce selection bias. In addition, our relatively small sample size may not have adequate power to detect incremental values of other biochemical parameters, such as hs-CRP, within this study. While our study population was heterogeneous, and the study was agnostic as to the interval therapy received by the patients (carried out at the discretion of the treating physician), this potential limitation is also a strength, highlighting the robustness of sST2, irrespective of the heterogeneity of the patient population or intercurrent treatments.

## 5. Conclusions

In conclusion, in patients with various cardiovascular diseases, sST2 levels are a robust determinant of hospital readmission due to MACE within 1 year. These results highlight the potential value of measuring sST2 levels for patients with a variety of CV diseases—not exclusively those with heart failure or acute cardiac ischemia. Larger population studies over longer period are required to fully elucidate the prognostic role of sST2 in predicting hospitalization due to MACE. These types of studies would also make it possible to explore the potential role of sST2 in predicting the response to specific treatments for CVD, such as statins, antiplatelet agents, or guideline-directed pillar classes of anti-heart failure therapies [37]. At the same time, further fundamental in vitro and in vivo studies are needed to better understand the mechanistic role of the IL-33/sST2 axis and how it contributes to the pathogenesis of CVD. This could allow for the further refinement and development of new therapeutic strategies targeting sST2 or its downstream signaling pathways.

## Figures and Tables

**Table 1:** Baseline patient characteristics.

Variable	Total (n = 250)
Age (years), mean ± SD ^1^	66 ± 17
Gender male/female, n (%)	158(63%)/92(37%)
Body mass index, (median, IQR ^2^)	28 (16–60)
**Co-morbidities**	
Hypertension, n (%)	119 (48%)
Dyslipidaemia, n (%)	76 (30%)
Heart failure, n (%)	69 (28%)
Diabetes Mellitus, n (%)	67 (27%)
Ischemic heart disease ^3^, n (%)	222 (89%)
Atrial Fibrillation, n (%)	44 (18%)
**MACE**	
Any MACE (≥1)	125 (50%)
Multiple MACE (≥2)	58 (23%)
**Medication**	
Statins, n (%)	188 (75%)
Antiplatelets, n (%)	175 (70%)
Beta-blockers, n (%)	171 (68%)
ACEi/ARB/ARNI n (%)	148 (60%)
**Biochemistry**	
Haemoglobin (g/L, median, min-max)	131 ± 20
Creatinine (µmol/L, median, min-max)	80 (27–755)
Estimated glomerular filtration rate (mL/min/1.73 m^2^, median, min-max)	81 (5–91)
C-reactive protein (mg/L, median, min-max)	6.5 (1–218)
Urea (mmol/L, median, min-max)	6 (1.4–70)
Soluble ST2 (ng/mL, median, min-max)	16.7 (1–78)
LDL ^4^ (mmol/L, mean, SD)	2.5 ± 0.9
HDL ^5^ (mmol/L, mean, SD)	1 ± 0.4

^1^ Standard deviation; ^2^ interquartile range; ^3^ IHD; ^4^ low density lipoprotein; ^5^ high density lipoprotein. IQR—interquartile ranges.

**Table 2:** Univariate correlates of categorical variables and sST2 levels.

Parameter	sST2 (ng/mL) (Median (Min, Max))	*p*
**Gender**		
Female	15.4 (1.0, 73.9)	0.03
Male	17.7 (3.0, 78.4)	
**Heart Failure**		
Yes	25.4 (5.4, 78.4)	<0.001
No	15.4 (1.0, 77.2)	
**Hypertension**		
Yes	17.9 (5.4, 74.3)	0.2
No	15.8 (1.0, 78.4)	
**Atrial Fibrillation**		
Yes	25.1 (3.0, 77.2)	0.01
No	15.9 (1.0, 78.4)	
**Diabetes mellitus**		
Yes	20.4 (5.7, 74.3)	0.2
No	16.1 (1.0, 78.4)	
**Ischemic heart disease**		
Yes	16.4 (1.0, 78.4)	0.4
No	20.7 (6.3, 53.0)	
**Dyslipidemia**		
Yes	16.4 (1.5, 77.2)	0.7
No	16.8 (1.0, 78.4)	
**Statin therapy**		
Yes	16.0 (1.0, 77.0)	0.1
No	20.3 (5.7, 78.4)	
**Antiplatelets**		
Yes	15.5 (1.0–78.4)	0.027
No	22.0 (4.9–77.2)	
**Beta-blockers**		
Yes	17.1 (1.0–78.4)	0.1
No	15.5 (2.8–77.2)	
**ACEi/ARB/ARNI ^1^**		
Yes	16.5 (1.5, 77.0)	0.6
No	17.1 (1.0, 78.4)	
**Any hospital admissions due to MACE (≥1)**		
Yes	19.5 (1.0–77.2)	0.1
No	15.6 (2.8, 78.4)	
**Hospital readmissions due to MACE (≥2)**		
Yes	23.8 (3.9–74.3)	0.047
No	16.2 (1.0–78.4)	

^1^ Angiotensin-converting enzyme inhibitors/angiotensin II receptor blockers/angiotensin II receptor blocker-neprilysin inhibitor.

**Table 3:** Univariate correlates of continuous variables and sST2 levels.

	sST2	
Variables	Spearman’s r	*p*
Age (years)	0.23	<0.001
BMI (kg/m^2^)	−0.14	0.1
Glucose (mmol/L)	0.01	0.9
Urea (mmol/L)	0.15	0.019
eGFR ^1^ (mL/min/1.73 m^2^)	−0.14	0.033
hs-CRP ^2^ (mg/L)	0.46	<0.001
Cholesterol (mmol/L)	−0.02	0.8
Triglycerides (mmol/L)	−0.05	0.5
LDL (mmol/L)	−0.03	0.7
HDL (mmol/L)	0.05	0.5

^1^ Estimated glomerular filtration rate; ^2^ high sensitive-C-reactive protein.

**Table 4:** Comparison of biochemistry levels between patients with or without MACE (statistics: Student’s *t*-test or Mann–Whitney test, as appropriate).

Variables	Patients without MACE (n = 125)	Patients with Any MACE (n = 125)	*p*
sST2 (ng/mL)	15.6 (1.0–78.4)	19.5 (1.0–77.2)	0.1
Age (years)	64 ± 13.0	71.8 (32–89)	0.024
BMI (kg/m^2^)	28.7 (16.0–55.1)	28.2 (16.0–60.3)	0.3
Hb (g/L)	132.7 ± 19.7	128.7 ± 20.6	0.1
Urea (mmol/L)	5.6 (1.4–70)	6.0 (2.6–36.8)	0.1
Creatinine (µmol/L)	75 (48–165)	82 (27–755)	0.1
eGFR (mL/min/1.73 m^2^)	84 (11–91)	80 (5–91)	0.1
Glucose (mmol/L)	5.7 (3.8–11.7)	5.7 (3.5–29.4)	0.6
TnI (ng/L)	86.5 (1.9–328726)	40 (1.9–233453)	0.4
CRP (mg/L)	6.5 (0.09–206)	6.0 (0.3- 218)	0.6
Cholesterol (mmol/L)	4.2 ±1.0	4.0 ± 1.1	0.2
Triglycerides (mmol/L)	1.1 (0.4–3.5)	1.2 (0.6–11.8)	0.3
LDL (mmol/L)	2.5 ± 0.9	2.4 ± 0.9	0.4
HDL (mmol/L)	1.0 (0.5–3.5)	0.9 (0.4–1.9)	0.04

**Table 5:** Patient’s characteristics and biochemistry levels comparisons by sST2 quartiles (statistics: Kruskal–Wallis H test or Chi-square test, as appropriate).

Variables	sST2 Q1 n = 62 (<11.1 ng/mL)	sST2 Q2 n = 63 (11.1–16.8 ng/mL)	sST2 Q3 n = 63 (16.8–28.4 ng/mL)	sST2 Q4 n = 62 (>28.4 ng/mL)	*p*
Gender(male/female)	32(52%)/30 (48%)	41(65%)/22 (35%)	41(65%)/22 (35%)	44(71%)/18 (29%)	0.1
IHD	55 (89%)	58 (92%)	57 (90%)	52 (84%)	0.5
Hypertension	21 (34%)	34 (54%)	33 (52%)	31 (50%)	0.1
Dyslipidaemia	14 (23%)	24 (38%)	21 (33%)	17 (27%)	0.3
Atrial Fibrillation	7 (11%)	8 (13%)	11 (17%)	18 (29%)	0.04
Heart Failure	10 (16%)	12 (19%)	21 (33%)	26 (42%)	0.003
Diabetes	14 (23%)	14 (22%)	19 (30%)	20 (32%)	0.5
Age (years)	60 ± 12.3	65 ± 13.3	68 ± 13.3	70 ± 13.3	0.001
BMI (kg/m^2^)	30.5 ± 7.0	29.4 ± 6.9	29.8 ± 7.3	28.7 ± 6.6	0.6
Hb (g/L)	136.2 ± 20.0	133.6 ± 23.8	127.3 ± 16.6	125.9 ± 18.4	0.01
Creatinine (µmol/L)	78.1 ± 21.1	82.0 (54–240)	77 (46–695)	82.5 (45–755)	0.1
eGFR (mL/min/1.73 m^2^)	86.5 (8–91)	82 (22–91)	79 (6–91)	76.5 (5–91)	0.048
Glucose (mmol/L)	5.6 (4.2–12.3)	5.8 (4.3–11.0)	5.7 (3.5–29.4)	5.8 (4.6–13.0)	0.6
TnI (ng/L)	31 (1.9–12620)	208 (1.9–233453)	15.0 (1.9–328726)	149 (2.0–213174)	0.2
hs-CRP (mg/L)	2.9 (0.3–24)	4.4 (0.3–74)	6.0 (0.1–206)	18 (2.0–218)	<0.001
Cholesterol (mmol/L)	4.1 ± 1.0	4.3 ± 1.1	4.1 ± 0.9	4.1 ± 1.3	0.7
Triglycerides (mmol/L)	1.2 (0.5–4.3)	1.2 (0.5–4.4)	1.2 (0.4–3.5)	1.2 (0.6–11.8)	0.6
LDL (mmol/L)	2.5 ± 1.0	2.6 ± 0.9	2.4 ± 0.8	2.3 ± 0.8	0.6
HDL (mmol/L)	0.9 (0.5–1.9)	1.0 (0.6–1.8)	1.1 (0.6–2.0)	0.9 (0.4–3.5)	0.5
Insulin (mmol/L)	10.6 (3.0–229)	10.4 (2.9–51.6)	8.0 (1.9–51.4)	9.8 (1.8–43.8)	0.5

**Table 6:** Adjusted multivariate analysis for risk factors associated with any MACE.

Variables	Exp Beta	95% C.I. (Lower, Upper)	*p*
sST2	1.02	(1.0–1.03)	0.045
Diabetes mellitus	2.88	(1.6–5.2)	0.001

Adjusted for: sST2 levels, sex, age, BMI, HT, DM, AF, HF, IHD and dyslipidaemia, statins, antiplatelets, beta-blockers, ACEi/ARB/ARNI.

**Table 7:** Adjusted multivariate analysis for risk factors associated with high levels of sST2 (fourth quartile).

Variables	Exp Beta	95% C.I. (Lower, Upper)	*p*
HF	1.77	(0.9–3.4)	0.087
Age	1.03	(1.0–1.1)	0.022
Statin	0.51	(0.2–1.1)	0.071
Beta-blockers	2.05	(1.1–4.8)	0.033
Number of MACE	1.35	(1.1–1.7)	0.007

Adjusted for: sex, age, BMI, HT, DM, AF, HF, IHD and dyslipidemia, statins, antiplatelets, beta-blockers, ACEi/ARB/ARNI.

## Data Availability

All data were collected via Hunter New England Health systems. All collected data are available upon request.

## Supplementary Materials

The following supporting information can be downloaded at: https://www.mdpi.com/article/10.3390/jcm12082790/s1, Table S1: Incidence of MACE by co-morbidity. Supplementary Figure S1: Comparison of sST2 levels in patients with or without comorbidities.
