# Supplementary material for: Elevated Soluble Suppressor of Tumorigenicity 2 Predict Hospital Admissions Due to Major Adverse Cardiovascular Events (MACE)
Source: J Clin Med. 2023 Apr 9;12(8):2790. doi: 10.3390/jcm12082790 (PMC10142832; doi:10.3390/jcm12082790)
Supplement: Supplementary file 1 [file jcm-12-02790-s001.zip › jcm-2265552-supplementary.pdf]

Supplementary Table S1. Incidence of MACE by co-morbidity

Co-morbidities	Incidence in total cohort (n=250)	MACE incidence amongst those with the comorbidity n (%)	P value
Hypertension, n (%)	119 (48%)	67 (56%)	0.076
Dyslipidemia, n (%)	76 (30%)	43 (57%)	0.216
Heart failure, n (%)	69 (28%)	39 (57%)	0.258
Diabetes Mellitus, n (%)	67 (27%)	46 (67%)	0.001
Ischemic heart disease[3], n (%)	222 (89%)	113 (51%)	0.548
Atrial Fibrillation, n (%)	44 (18%)	23 (52%)	0.868

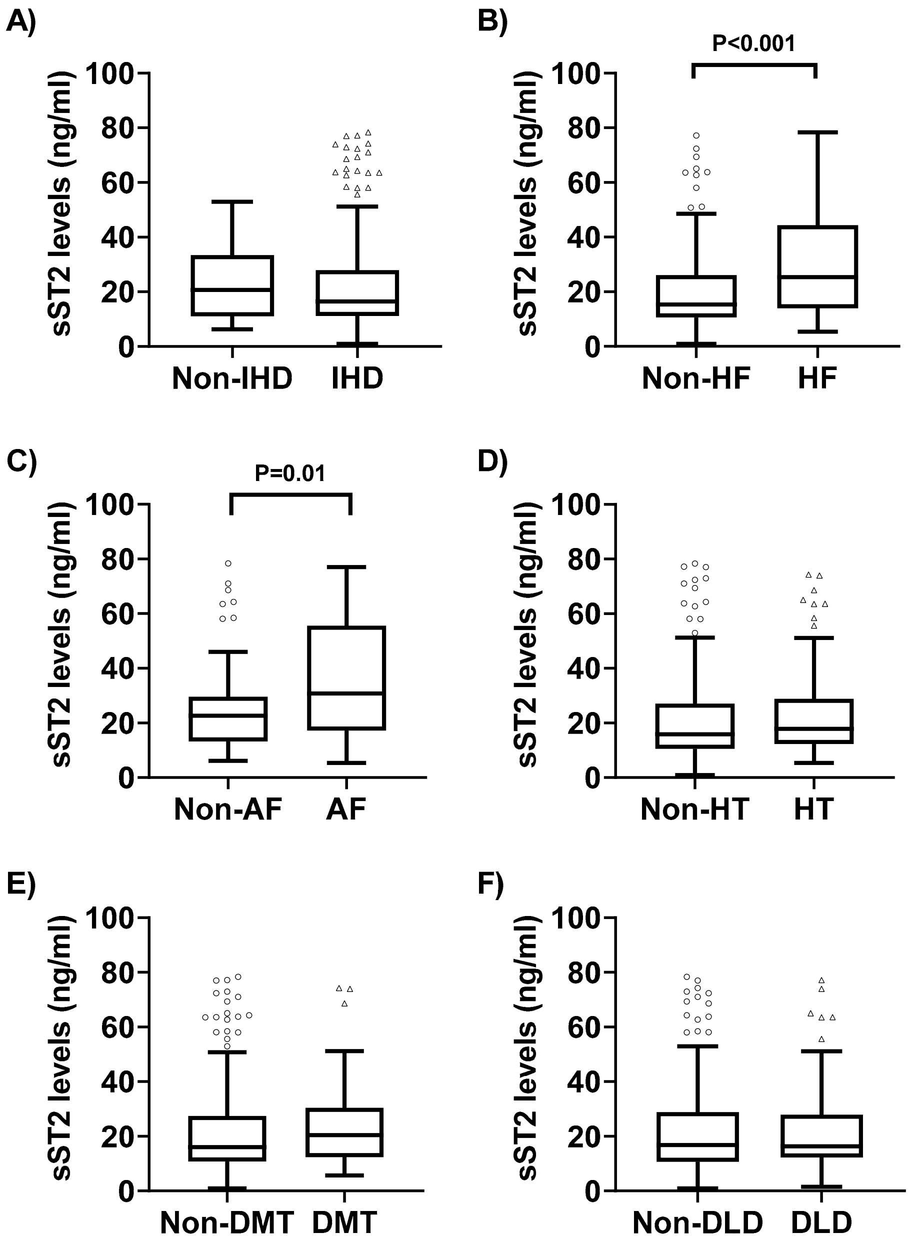

Supplementary Figure S1. Comparison of sST2 levels in patients with or without A) ischemic heart disease, B) heart failure, C) atrial fibrillation, D) hypertension, E) diabetes mellitus, F) dyslipidemia.
